# The effects of Fentanyl on executive cognitive function and mental health

**DOI:** 10.1192/j.eurpsy.2026.10480

**Published:** 2026-07-31

**Authors:** T. Peeri, Y. Mama, P. Roska, A. Weinstein

**Affiliations:** 1 Psychology, Ariel University, Ariel; 2 Treatment for substance abuse, Ministry of Health, Jerusalem, Israel

## Abstract

**Introduction:**

Synthetic opioids such as Fentanyl have effects similar to natural therapeutic opioids. The new Fentanyl is a narcotic drug, commonly used as a surgical anesthetic because of its high potency. Therefore, its use often results in various adverse effects such as overdose and death. Few studies have investigated the chronic long-term effects of opiate use on cognitive function and the brain. It has been suggested that the mu-opioid system can influence higher-level cognitive function by modulating evaluation, motivation, and control circuits that are densely populated with mu-opioid receptors, including the orbitofrontal cortex, basal ganglia, amygdala, anterior cingulate cortex, and prefrontal cortex. It has also been proposed that opioids influence decision-making and cognitive control by increasing the subjective value of reward and reducing aversive arousal. Given the paucity in research on the effects of Fentanyl on cognitive function, there is an urgent need to investigate cognitive function and mental health in synthetic Fentanyl abusers.

**Objectives:**

Our study aimed to investigate the effects of chronic use of fentanyl on executive function and mental health in Fentanyl users and healthy control participants.

**Methods:**

Participants- A total of 11 Fentanyl users and 19 healthy non-user participants. inclusion criteria for Fentanyl users were chronic use of SF, age 18+ years, and age at injury <45 years who had used Fentanyl for at least 1 year before enrollment in the study.

Questionnaires- Demographic and substance use history, Beck Depression Inventory, Spielberger state-trait anxiety inventory.

Computerized tasks- Working memory – The N-back task, Inhibitory control – The Stroop word-color task, Inhibitory control – Go/No-Go task, Long-term Memory free-recall task.

**Results:**

Fentanyl users exhibited impaired executive function on the n-back task compared with control participants. They were less accurate and they showed longer reaction times on the 2-back and 1-back tasks compared to control participants. They were also slower on the Go/No-Go task and showed more commission and omission errors than control participants, indicating impaired inhibition. They also responded longer and made more errors on the Stroop task, indicating impaired attention. They remembered fewer words on a long-term memory task than control participants. Finally, Fentanyl users scored higher on the Beck Depression Inventory (BDI) and the Spielberger State-Trait Anxiety Inventory (STAI).

**Conclusions:**

This study showed significant cognitive impairments in fentanyl users compared with healthy non-users, highlighting the potential long-term consequences of fentanyl use. These findings underscore the importance of addressing cognitive decline in this population, which may have major implications for their daily functioning and overall quality of life.

**Disclosure of Interest:**

None Declared

